# Anilinium 3-(4-hy­droxy-3-meth­oxy­phenyl)prop-2-enoate

**DOI:** 10.1107/S1600536810043412

**Published:** 2010-10-31

**Authors:** Li-Cai Zhu

**Affiliations:** a School of Chemistry and Environment, South China Normal University, Guangzhou 510631, People’s Republic of China

## Abstract

The structure of the title salt, C_6_H_8_N^+^·C_10_H_9_O_4_^−^, is stabilized by N—H⋯O and O—H⋯O hydrogen bonding between 3-(4-hy­droxy-3-meth­oxy­phen­yl)prop-2-enoate anions and anilinium cations, which links the components into a two-dimensional array.

## Related literature

For ferulic acid [3-(4-hy­droxy-3-meth­oxy­phen­yl)-2-propenoic acid] and its pharmacological activity, see: Hirabayashi *et al.* (1995 ▶); Liyama *et al.* (1994 ▶); Nomura *et al.* (2003 ▶); Ogiwara *et al.* (2002 ▶); Ou *et al.* (2003 ▶). For crystal structures on hydrogen-bond motifs in organic ammonium salts, see: Ni *et al.* (2007 ▶); Smith *et al.* (2004 ▶).
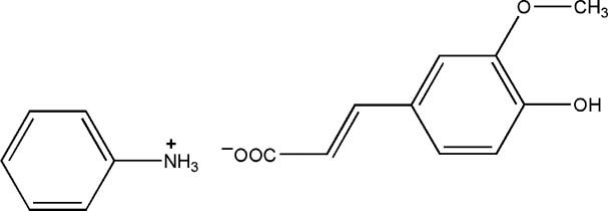


## Experimental

### 

#### Crystal data

C_6_H_8_N^+^·C_10_H_9_O_4_^−^
*M* *_r_* = 287.31
Orthorhombic, 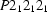

*a* = 6.2047 (7) Å
*b* = 8.2668 (9) Å
*c* = 28.790 (3) Å
*V* = 1476.7 (3) Å^3^
*Z* = 4
Mo *K*α radiation
μ = 0.09 mm^−1^
*T* = 296 K
0.30 × 0.28 × 0.25 mm

#### Data collection

Bruker APEXII area-detector diffractometer
7666 measured reflections
1586 independent reflections
1325 reflections with *I* > 2σ(*I*)
*R*_int_ = 0.031

#### Refinement

*R*[*F* ^2^ > 2σ(*F* ^2^)] = 0.032
*wR*(*F* ^2^) = 0.079
*S* = 1.04
1586 reflections
193 parameters
H-atom parameters constrained
Δρ_max_ = 0.11 e Å^−3^
Δρ_min_ = −0.11 e Å^−3^

### 

Data collection: *APEX2* (Bruker, 2004 ▶); cell refinement: *SAINT* (Bruker, 2004 ▶); data reduction: *SAINT*; program(s) used to solve structure: *SHELXS97* (Sheldrick, 2008 ▶); program(s) used to refine structure: *SHELXL97* (Sheldrick, 2008 ▶); molecular graphics: *SHELXTL* (Sheldrick, 2008 ▶); software used to prepare material for publication: *SHELXL97*.

## Supplementary Material

Crystal structure: contains datablocks I, global. DOI: 10.1107/S1600536810043412/om2374sup1.cif

Structure factors: contains datablocks I. DOI: 10.1107/S1600536810043412/om2374Isup2.hkl

Additional supplementary materials:  crystallographic information; 3D view; checkCIF report

## Figures and Tables

**Table 1:** Hydrogen-bond geometry (Å, °)

*D*—H⋯*A*	*D*—H	H⋯*A*	*D*⋯*A*	*D*—H⋯*A*
N1—H1*A*⋯O2^i^	0.89	1.84	2.721 (2)	170
N1—H1*B*⋯O1^ii^	0.89	1.84	2.724 (2)	170
N1—H1*C*⋯O2	0.89	1.85	2.730 (3)	170
O3—H3*A*⋯O1^iii^	0.82	2.09	2.841 (2)	151
O3—H3*A*⋯O4	0.82	2.25	2.671 (2)	112

Symmetry codes: (i) 
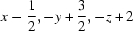
; (ii) 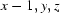; (iii) 
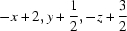
.

**Fig. 1.**
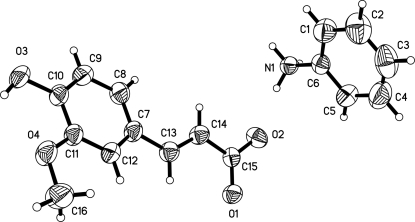
The molecular structure showing the atomic-numbering scheme and displacement ellipsoids drawn at the 50% probability level.

**Fig. 2.**
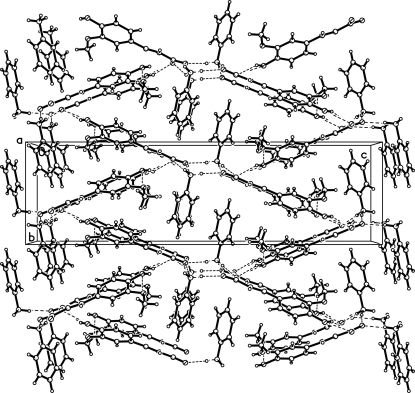
The molecular packing showing the hydrogen-bonding interactions as broken lines.

## supplementary crystallographic information

### Comment

3-(4-Hydroxy-3-methoxyphenyl)-2-propenoic acid, also known as ferulic acid, is one of the main endogenous phenolic acids in plant kingdom (Liyama *et al.*, 1994). More attention was paid to the structural modification of ferulic acid owing to its extensive bioactivities including anti-platelet aggregation, anti-oxidation, anti-inflammation, anti-tumor, anti-mutagenicity, antibiosis and immunity enchancement (Hirabayashi *et al.*, 1995; Ogiwara *et al.*, 2002). A series of ferulic acid derivatives were designed and synthesized, such as their salts, esters, ethers and amides, and some of them show the better bioactivities than those of ferulic acid (Nomura *et al.*, 2003; Ou *et al.*, 2003). The molecular and crystal structure of the title compound is presented in this article.

In the asymmetric unit of the title compound, illustrated in Fig. 1, there are an anilinium cation and one singly deprotonated 3-(4-hydroxy-3-methoxyphenyl)prop-2-enoate anion. The bond distances and angles in the title compound are normal (Ni *et al.*, 2007; Smith *et al.*, 2004). In the crystal the cations and anions are self-assembled by various O—H···O and N—H···O hydrogen bonds (Table 1 and Fig. 2) to form a superamolecular network. The network can be viewed as the linkages of two-dimensional 3-(4-hydroxy-3-methoxyphenyl)prop-2-enoate and anilinium layers.

### Experimental

A mixture of ferulic acid (0.388 g, 2 mmol) and aniline (0.19 ml, 2 mmol) was stirred with methanol (20 ml) for 0.5 h at room temperature. After several days colourless block-like crystals, suitable for X-ray diffraction analysis, were obtained by slow evaporation of the solution.

### Refinement

All H atoms were placed at calculated positions and were treated as riding, with C—H = 0.93–0.96 Å, O—H = 0.82 Å, and N—H = 0.89 Å, respectively, and with *U*_iso_(H) = 1.2 or 1.5*U*_eq_(C,O,N).

### Crystal data

**Table:**

C_6_H_8_N^+^·C_10_H_9_O_4_^−^	*F*(000) = 608
*M**_r_* = 287.31	*D*_x_ = 1.292 Mg m^−^^3^
Orthorhombic, *P*2_1_2_1_2_1_	Mo *K*α radiation, λ = 0.71073 Å
Hall symbol: P 2ac 2ab	Cell parameters from 2062 reflections
*a* = 6.2047 (7) Å	θ = 2.6–23.1°
*b* = 8.2668 (9) Å	µ = 0.09 mm^−^^1^
*c* = 28.790 (3) Å	*T* = 296 K
*V* = 1476.7 (3) Å^3^	Block, colourless
*Z* = 4	0.30 × 0.28 × 0.25 mm

### Data collection

**Table:**

Bruker APEXII area-detector diffractometer	1325 reflections with *I* > 2σ(*I*)
Radiation source: fine-focus sealed tube	*R*_int_ = 0.031
graphite	θ_max_ = 25.2°, θ_min_ = 2.6°
φ and ω scans	*h* = −7→7
7666 measured reflections	*k* = −9→6
1586 independent reflections	*l* = −32→34

### Refinement

**Table:**

Refinement on *F*^2^	Primary atom site location: structure-invariant direct methods
Least-squares matrix: full	Secondary atom site location: difference Fourier map
*R*[*F*^2^ > 2σ(*F*^2^)] = 0.032	Hydrogen site location: inferred from neighbouring sites
*wR*(*F*^2^) = 0.079	H-atom parameters constrained
*S* = 1.04	*w* = 1/[σ^2^(*F*_o_^2^) + (0.0375*P*)^2^ + 0.170*P*] where *P* = (*F*_o_^2^ + 2*F*_c_^2^)/3
1586 reflections	(Δ/σ)_max_ = 0.001
193 parameters	Δρ_max_ = 0.11 e Å^−^^3^
0 restraints	Δρ_min_ = −0.11 e Å^−^^3^

### Special details

**Table:**

Geometry. All e.s.d.'s (except the e.s.d. in the dihedral angle between two l.s. planes) are estimated using the full covariance matrix. The cell e.s.d.'s are taken into account individually in the estimation of e.s.d.'s in distances, angles and torsion angles; correlations between e.s.d.'s in cell parameters are only used when they are defined by crystal symmetry. An approximate (isotropic) treatment of cell e.s.d.'s is used for estimating e.s.d.'s involving l.s. planes.
Refinement. Refinement of *F*^2^ against ALL reflections. The weighted *R*-factor *wR* and goodness of fit *S* are based on *F*^2^, conventional *R*-factors *R* are based on *F*, with *F* set to zero for negative *F*^2^. The threshold expression of *F*^2^ > σ(*F*^2^) is used only for calculating *R*-factors(gt) *etc*. and is not relevant to the choice of reflections for refinement. *R*-factors based on *F*^2^ are statistically about twice as large as those based on *F*, and *R*- factors based on ALL data will be even larger.

### Fractional atomic coordinates and isotropic or equivalent isotropic displacement parameters (Å^2^)

**Table:**

	*x*	*y*	*z*	*U*_iso_*/*U*_eq_	
C1	0.3231 (5)	0.4376 (3)	0.92787 (9)	0.0626 (7)	
H1	0.2083	0.5019	0.9183	0.075*	
C2	0.3238 (7)	0.2733 (4)	0.91830 (10)	0.0799 (10)	
H2	0.2092	0.2272	0.9022	0.096*	
C3	0.4919 (7)	0.1789 (4)	0.93232 (10)	0.0794 (10)	
H3	0.4919	0.0687	0.9258	0.095*	
C4	0.6609 (7)	0.2469 (4)	0.95602 (11)	0.0792 (9)	
H4	0.7752	0.1823	0.9657	0.095*	
C5	0.6628 (5)	0.4108 (3)	0.96573 (9)	0.0609 (7)	
H5	0.7778	0.4570	0.9817	0.073*	
C6	0.4920 (4)	0.5044 (3)	0.95139 (7)	0.0422 (5)	
C7	0.8501 (4)	1.0202 (3)	0.79047 (7)	0.0434 (6)	
C8	0.6513 (4)	1.0959 (3)	0.79226 (7)	0.0494 (6)	
H8	0.5777	1.1016	0.8204	0.059*	
C9	0.5597 (4)	1.1639 (3)	0.75247 (8)	0.0509 (6)	
H9	0.4288	1.2185	0.7543	0.061*	
C10	0.6649 (4)	1.1496 (3)	0.71042 (7)	0.0447 (6)	
C11	0.8608 (4)	1.0664 (3)	0.70800 (7)	0.0417 (6)	
C12	0.9540 (4)	1.0060 (3)	0.74787 (8)	0.0443 (6)	
H12	1.0876	0.9553	0.7463	0.053*	
C13	0.9606 (4)	0.9557 (3)	0.83187 (7)	0.0451 (6)	
H13	1.1098	0.9473	0.8299	0.054*	
C14	0.8738 (4)	0.9089 (3)	0.87121 (7)	0.0437 (6)	
H14	0.7250	0.9167	0.8744	0.052*	
C15	0.9992 (4)	0.8447 (3)	0.91045 (7)	0.0375 (5)	
C16	1.1413 (5)	0.9702 (4)	0.65865 (9)	0.0711 (9)	
H16A	1.1252	0.8615	0.6698	0.107*	
H16B	1.2524	1.0238	0.6760	0.107*	
H16C	1.1798	0.9681	0.6264	0.107*	
N1	0.4930 (3)	0.6767 (2)	0.96199 (5)	0.0411 (5)	
H1A	0.4750	0.6908	0.9924	0.062*	
H1B	0.3863	0.7252	0.9467	0.062*	
H1C	0.6184	0.7195	0.9533	0.062*	
O1	1.2000 (3)	0.8328 (2)	0.90760 (5)	0.0467 (4)	
O2	0.8938 (3)	0.8013 (2)	0.94615 (5)	0.0536 (5)	
O3	0.5753 (3)	1.2193 (3)	0.67198 (5)	0.0578 (5)	
H3A	0.6647	1.2222	0.6510	0.087*	
O4	0.9441 (3)	1.0547 (2)	0.66423 (5)	0.0535 (5)	

### Atomic displacement parameters (Å^2^)

**Table:**

	*U*^11^	*U*^22^	*U*^33^	*U*^12^	*U*^13^	*U*^23^
C1	0.0678 (18)	0.0590 (18)	0.0609 (16)	−0.0043 (16)	−0.0183 (15)	−0.0011 (14)
C2	0.105 (3)	0.0604 (19)	0.0737 (19)	−0.014 (2)	−0.012 (2)	−0.0107 (17)
C3	0.116 (3)	0.0483 (17)	0.074 (2)	0.002 (2)	0.025 (2)	−0.0048 (16)
C4	0.084 (2)	0.063 (2)	0.091 (2)	0.0227 (19)	0.013 (2)	0.0134 (17)
C5	0.0573 (18)	0.0609 (18)	0.0644 (16)	0.0078 (16)	−0.0051 (14)	0.0067 (15)
C6	0.0482 (14)	0.0475 (14)	0.0309 (11)	0.0000 (12)	0.0042 (11)	0.0030 (10)
C7	0.0476 (14)	0.0472 (14)	0.0353 (11)	0.0003 (12)	−0.0062 (11)	0.0048 (10)
C8	0.0545 (16)	0.0578 (15)	0.0358 (12)	0.0016 (14)	0.0036 (12)	0.0035 (12)
C9	0.0449 (14)	0.0592 (16)	0.0486 (13)	0.0042 (13)	−0.0044 (12)	0.0063 (12)
C10	0.0461 (14)	0.0510 (14)	0.0370 (12)	−0.0037 (13)	−0.0093 (11)	0.0065 (11)
C11	0.0457 (14)	0.0442 (13)	0.0352 (11)	−0.0001 (12)	−0.0049 (10)	0.0044 (11)
C12	0.0443 (14)	0.0475 (14)	0.0411 (12)	0.0034 (12)	−0.0045 (10)	0.0058 (11)
C13	0.0459 (14)	0.0503 (14)	0.0391 (11)	0.0010 (12)	−0.0033 (11)	0.0009 (11)
C14	0.0428 (14)	0.0536 (14)	0.0348 (11)	0.0025 (12)	−0.0047 (10)	−0.0010 (11)
C15	0.0464 (14)	0.0374 (12)	0.0286 (11)	−0.0038 (12)	−0.0052 (10)	−0.0022 (9)
C16	0.0608 (19)	0.101 (2)	0.0509 (15)	0.0196 (19)	0.0077 (14)	0.0040 (16)
N1	0.0404 (10)	0.0512 (12)	0.0317 (9)	0.0005 (10)	−0.0024 (8)	0.0043 (8)
O1	0.0436 (10)	0.0584 (11)	0.0381 (8)	0.0026 (9)	−0.0049 (7)	0.0040 (8)
O2	0.0523 (10)	0.0775 (12)	0.0310 (8)	−0.0123 (10)	−0.0013 (7)	0.0068 (8)
O3	0.0490 (10)	0.0807 (12)	0.0439 (9)	0.0115 (10)	−0.0081 (8)	0.0188 (10)
O4	0.0577 (11)	0.0665 (11)	0.0364 (8)	0.0128 (10)	−0.0014 (8)	0.0083 (8)

### Geometric parameters (Å, °)

**Table:**

C1—C6	1.365 (4)	C10—O3	1.366 (3)
C1—C2	1.386 (4)	C10—C11	1.399 (3)
C1—H1	0.9300	C11—O4	1.365 (3)
C2—C3	1.363 (5)	C11—C12	1.379 (3)
C2—H2	0.9300	C12—H12	0.9300
C3—C4	1.372 (5)	C13—C14	1.313 (3)
C3—H3	0.9300	C13—H13	0.9300
C4—C5	1.384 (4)	C14—C15	1.470 (3)
C4—H4	0.9300	C14—H14	0.9300
C5—C6	1.375 (4)	C15—O1	1.253 (3)
C5—H5	0.9300	C15—O2	1.270 (3)
C6—N1	1.457 (3)	C16—O4	1.418 (3)
C7—C8	1.384 (4)	C16—H16A	0.9600
C7—C12	1.390 (3)	C16—H16B	0.9600
C7—C13	1.475 (3)	C16—H16C	0.9600
C8—C9	1.397 (3)	N1—H1A	0.8900
C8—H8	0.9300	N1—H1B	0.8900
C9—C10	1.381 (3)	N1—H1C	0.8900
C9—H9	0.9300	O3—H3A	0.8200
			
C6—C1—C2	119.5 (3)	O4—C11—C12	125.8 (2)
C6—C1—H1	120.3	O4—C11—C10	114.18 (19)
C2—C1—H1	120.3	C12—C11—C10	120.1 (2)
C3—C2—C1	120.3 (3)	C11—C12—C7	120.6 (2)
C3—C2—H2	119.8	C11—C12—H12	119.7
C1—C2—H2	119.8	C7—C12—H12	119.7
C2—C3—C4	119.8 (3)	C14—C13—C7	127.8 (2)
C2—C3—H3	120.1	C14—C13—H13	116.1
C4—C3—H3	120.1	C7—C13—H13	116.1
C3—C4—C5	120.6 (3)	C13—C14—C15	123.5 (2)
C3—C4—H4	119.7	C13—C14—H14	118.2
C5—C4—H4	119.7	C15—C14—H14	118.2
C6—C5—C4	118.9 (3)	O1—C15—O2	122.9 (2)
C6—C5—H5	120.6	O1—C15—C14	120.3 (2)
C4—C5—H5	120.6	O2—C15—C14	116.8 (2)
C1—C6—C5	120.9 (2)	O4—C16—H16A	109.5
C1—C6—N1	120.2 (2)	O4—C16—H16B	109.5
C5—C6—N1	118.9 (2)	H16A—C16—H16B	109.5
C8—C7—C12	119.0 (2)	O4—C16—H16C	109.5
C8—C7—C13	123.2 (2)	H16A—C16—H16C	109.5
C12—C7—C13	117.8 (2)	H16B—C16—H16C	109.5
C7—C8—C9	120.9 (2)	C6—N1—H1A	109.5
C7—C8—H8	119.5	C6—N1—H1B	109.5
C9—C8—H8	119.5	H1A—N1—H1B	109.5
C10—C9—C8	119.5 (2)	C6—N1—H1C	109.5
C10—C9—H9	120.3	H1A—N1—H1C	109.5
C8—C9—H9	120.3	H1B—N1—H1C	109.5
O3—C10—C9	118.8 (2)	C10—O3—H3A	109.5
O3—C10—C11	121.4 (2)	C11—O4—C16	117.76 (18)
C9—C10—C11	119.8 (2)		

### Hydrogen-bond geometry (Å, °)

**Table:**

*D*—H···*A*	*D*—H	H···*A*	*D*···*A*	*D*—H···*A*
N1—H1A···O2^i^	0.89	1.84	2.721 (2)	170
N1—H1B···O1^ii^	0.89	1.84	2.724 (2)	170
N1—H1C···O2	0.89	1.85	2.730 (3)	170
O3—H3A···O1^iii^	0.82	2.09	2.841 (2)	151
O3—H3A···O4	0.82	2.25	2.671 (2)	112

Symmetry codes: (i) *x*−1/2, −*y*+3/2, −*z*+2; (ii) *x*−1, *y*, *z*; (iii) −*x*+2, *y*+1/2, −*z*+3/2.
